# Spectroscopic Assessment of Normal Cortical Bone: Differences in Relation to Bone Site and Sex

**DOI:** 10.1100/tsw.2010.43

**Published:** 2010-03-05

**Authors:** Nikolaos Kourkoumelis, Margaret Tzaphlidou

**Affiliations:** Department of Medical Physics, Medical School, University of Ioannina, Greece

**Keywords:** Fourier transform infrared spectroscopy (FTIR), vibrational spectroscopy, energy-dispersive X-ray spectroscopy (EDX), SEM, bone mineral, cortical bone, Ca/P ratio

## Abstract

Bone is a highly complex, composite tissue and its properties normally vary with age, type, and disorders. Fourier transform infrared spectroscopy and energy-dispersive X-ray spectroscopy techniques were used to study the effect of bone sites and sex to mineral and matrix content and composition. The results show that in rats, all inorganic phases consist of poorly crystalline B-type carbonated apatite, while overall mineralization and carbonate content is virtually unaffected among samples. Statistically significant differences were detected for the nonapatitic environments of acid phosphate and carbonate content. The mean values for the Ca/P ratio point to an increasing trend from tibia to forearm, and to femoral sections.

### INTRODUCTION

Bone, at the molecular level, is described as a composite material consisting of calcium phosphate crystals, water, and soft organic material, mostly collagen, which surrounds the mineral crystals by an exceptionally dense filling[1]. These constituents are commonly referred to as mineral and matrix components, and are distributed in diverse patterns among different bone types. The mineral phase in calcified tissues plays a significant role, mainly because it strongly affects their strength and quality. Qualitative parameters, such as mineral crystal perfection, composition, and size, vary considerably in relation to bone age, type, site, and also to bone metabolic diseases. Mechanical strength mainly depends on the state of cortical bone[2]. Stress tests on excised femoral necks have shown that the cortex contributes 40–60% of the overall strength[3]. In addition, studies on stress distributions associated with normal gait showed that the majority of load in the femoral neck region is carried by cortical bone[4]. Thus, in the present work, the effect of bone site and sex on mineral content and composition of rat cortical bone was studied.

For the nondestructive examination of biological tissues, among other techniques, Fourier transform infrared (FTIR) spectroscopy is often used. This technique is particularly useful because it relies on objective criteria such as changes of the IR absorption frequency and intensity in various functional groups of biological molecules. For bone, FTIR spectroscopy can be used to scrutinize mineralization, maturity of mineral crystal and collagen, carbonate accumulation and environment, as well as acid phosphate content[5]. Phosphate, carbonate, and hydrogenophosphate ions have an important effect on the formation and maturation of bone mineral since their relative concentration indicates the stoichiometric evolution of poorly crystalline biological apatite. This evolution proceeds by the transformation of the initial, rich-in-nonapatitic-species phase into a stable, more crystalline, carbonate apatite.

The relative content of calcium against phosphorus is generally accepted as a regulator to maintain mineral homeostasis and bone metabolism[6]. It has been proposed that changes of the Ca/P ratio are more crucial for the valuation of bone health than variations of the concentrations of either Ca or P[7,8,9], as these deviations are not necessarily in mutual association. Thus, a method able to access the Ca/P ratio could lead to a greater understanding of the role played by these elements. Energy-dispersive X-ray (EDX) spectroscopy is a sensitive qualitative and semi-quantitative procedure for the assessment of the mineral content variations in microscopic regions of bone[10]. Furthermore, the range of Ca/P values in response to the parameters studied can be correlated with the spectroscopic results.

### MATERIALS AND METHODS

#### Animals – Bone Samples

Ten female and male Wistar rats, 8 months of age, were used for the present study. Animals were housed and bred in natural conditions and euthanized under light ether anesthesia. Cortical sections from three different bone sites, femur, tibia, and forearm (ulna), all from the right side, were dissected out and cleaned free of soft tissue. There were no signs of metabolic alterations of the bone tissue. To facilitate removal of loosely bound water, the samples were freeze dried. All studies are approved by the Ioannina University Institutional Animal Care and Use Committee.

#### FTIR Spectral Data Collection

FTIR spectra were recorded on a Perkin-Elmer Spectrum GX FTIR system with samples prepared as KBr pellets, in the 4000- to 370-$cm^{-1}$ range, at a resolution of 4 $cm^{-1}$. The resolution of the spectra was retained, as no smoothing algorithms were applied prior to deconvolution analysis. Pellets were prepared by mixing KBr and bone powder (10% by weight) in a mortar and pestle, and pressed in a 13-mm pellet die. FTIR spectra were acquired immediately after lyophilization.

#### EDX Data Collection

The samples were examined by scanning electron microscopy (SEM) in a Jeol JSM 5600 system configured with an EDX detector operating at 20-kV accelerating voltage and 20-mm working distance. The EDX data were compiled for analysis using the Link ISIS system (Oxford Instruments, U.K.). The Ca/P ratio was calculated using the accompanying ZAF-correction software (SEMQuant, Oxford Instruments). A standard sample with similar Ca/P ratio was analyzed prior to each EDX session. Due to the fact that the scanning electron microscope operates at vacuum conditions, the tissue was partially dehydrated with an organic, water-miscible solvent. This was achieved by upgrading a 70% solution of EtOH to absolute in ascending concentrations. Then, the tissues were slightly polished, further dehydrated by freeze drying, and coated with a conductive carbon layer using a JEOL JEE-4X Vacuum Evaporator. The electron beam can induce significant damage on biological substances[11], so we performed excessive tests on operating conditions. Thus, the magnification was kept low (×50 – ×110) and each spectrum was collected twice every 60 sec until a total collection time of 120 sec was reached. No significant discrepancies were found between each measurement concerning the Ca/P ratio, which was finally estimated from the mean value of two different locations of the specimen.

### Data Analysis

Statistical analysis for the parameters under study was carried out utilizing Graph Pad INSTAT software (GraphPad Software, San Diego, CA). Data are reported as mean ± SD. Evaluation of statistical significance of differences of mean values was performed using nonparametric, one-way ANOVA (Kruskal-Wallis analysis of ranks) for the three bone site groups and Mann-Whitney *t*-test for the two populations. Even though the physical substrate of the experiment designates Gaussian distribution of the data, we followed rigid statistical methods owing to the small sample size. Statistical significance was considered at $p < 0.05$. Curve fitting and area integrations were performed using OriginPro (OriginLab Corporation, Northampton, MA) and the minimization was based on the Levenbeg-Marquardt algorithm. Band shapes were always considered as Gaussian and were baseline corrected. The convergence criterion was met in all cases with $R^2 = 0.99$. The computed area of each sub-band is reported as the percentage of the integrated area of the whole band. Spectra manipulation was done with ACD/Specmanager (ACD Labs, Toronto, Canada) and peak positions were originated by second derivative analysis using the Savitsky-Golay method (second-order polynomial with 19 data points).

## RESULTS AND DISCUSSION

### FTIR Spectra

A typical processed IR spectrum of the homogenized bone sample is depicted in Fig. 1, where regions of interest are also shown.

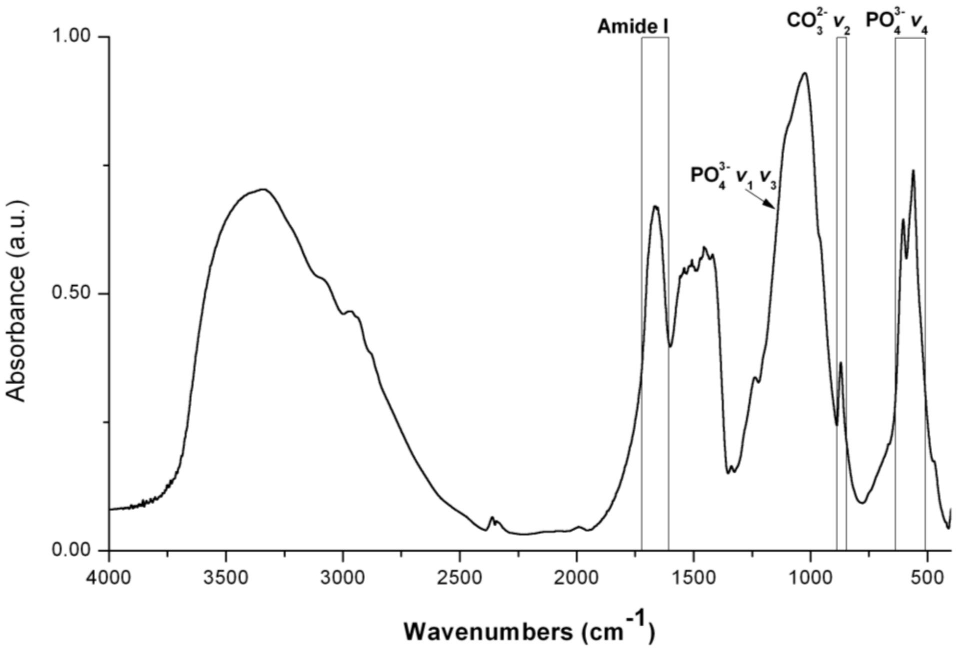

**FIGURE 1.** Typical IR spectra of homogenized bone samples. Regions of interest are indicated.

The measured spectra do not differ appreciably (±1 $cm^{-1}$) and, consequently, some common diagnostic vibrational bands are reported in Table 1.

**TABLE 1**
**Bands Assignments[12,13]**

| Assignment | IR Frequency ($cm^{-1}$) |
|---|---|
| $PO_4^{3-}$ $v_2$ | 473w |
| $PO_4^{3-}$ $v_4$ | 561s, 603s[a] |
| $CO_3^{2-}$ $v_4$ | 669vw |
| $CO_3^{2-}$ $v_2$ | 871s[a] |
| $PO_4^{3-}$ $v_1$ | 957sh |
| $HPO_4^{2-}$ $v_3$ | 1163vw |
| $PO_4^{3-}$ $v_3$ | 1027s, 1101w |
| Amide III | 1236s |
| $CH_2$ wagging | 1340s |
| $v COO^-$ | 1398s |
| $\delta_{as}CH_3$, $CO_3^{2-}$ $v_3$ | 1460s[b] |
| $v_{as}COO^-$ | 1510s[b] |
| Amide II δ(N-H) v(C-N) (C-N stretching and N-H in-plane bending) | 1546s–1562s |
| Amide I (C=O stretch) | 1649s |
| C=O nonionized | 1735w |
| $v CH_2$ | 2885sh |
| $v_{as}CH_2$ | 2938w |
| $v_{as}CH_3$ | 2963mw |
| *v*(C-H) Amide B | 3075w |
| *v*(O-H), *v*(N-H) (Amide A) | 3185s–3575s |

[a]IR envelope; [b]poorly resolvable.

In the functional group region, the intense band at 1649 $cm^{-1}$ is attributed to the absorption of the Amide I functional group (peptide C=O stretching vibration of the collagen), which represents the organic matrix of bone since the vast percentage of the protein content is collagen (mainly type I). It also shows that the main helix band is dominated by the secondary structure of collagen[14]. The Amide II band near 1550 $cm^{-1}$ is due to a combination of the C-N stretch and N-H in-plane bending modes. In the fingerprint region, the broad and strong absorption band at 900–1200 $cm^{-1}$ is assigned to the $v_1$ and $v_3$ normal modes of the apatitic phosphate ion, the most profuse ion in bone mineral. The free ion exhibits four fundamental modes of vibrations: $v_1$ ($A_1$) (symmetric stretching), $v_2$ (E) (symmetric bending), $v_3$ ($T_2$) (antisymmetric stretching), and $v_4$ ($T_2$) (antisymmetric bending)[15,16]. Due to the fact that only the triply degenerate vibration species of the tetrahedral molecules are IR active, only $v_3$ and $v_4$ vibration modes should be observed. Nevertheless, the other two modes, $v_1$ and $v_2$, become observable as the geometrical parameters shift to lower molecular symmetry. In our case, the symmetry of the phosphate ion has obviously been lowered to a point group compatible with $v_1$, $v_2$ modes; this is evident from the weak bands at 957 and 473 $cm^{-1}$, respectively. The appearance of the latent $v_1$ dipole, as well as the interaction with the neighboring atoms, results in the removal of the degeneracy of the $v_3$ mode. Thus, the wide range of 960–1140 $cm^{-1}$ may possibly be deconvoluted by a maximum of three absorption bands. The doublet at 561 and 603 $cm^{-1}$ is assigned to the $v_4$ bending mode. It is of significance to mention that no hydroxide ion bands appear in this region, typically as a weak shoulder at 630 $cm^{-1}$, as is the case with (mostly synthetic)

hydroxyapatites. This absence of the $OH^{-}$ band in the spectra of bone samples probably does not denote the complete deficiency of hydroxide ions in bone mineral. This is further supported by the data presented in Table 1, plus other studies[17,18]. The $v$(OH) band is shown in Fig. 1 as a wide contour and is most probably attributed to embedded water molecules and not to lattice hydroxyl ions. The lack or not of hydroxyl ions in bone apatite is still under investigation and a matter of dispute[19] among researchers. Given the number of bands as well as crystallographic data[20], we conclude that the phosphate ion of the biological apatite adopts the $C_{3v}$ point group symmetry instead of the $T_d$ symmetry of the free ion, giving three formal IR active vibrations. Further complications, such as $HPO_4^{2-}$ ions and the presence of vacancies in the lattice, expand the number of possible convoluted sub-bands of the wide phosphate moiety. The presence of the band at 1163 $cm^{-1}$ is attributed to the $HPO_4^{2-}$ related to nonstoichiometric hydroxyapatite. The latter and its content decrease have been connected with the high proportion of organic content in bone[17]. The very low intensity of this absorption band is consistent with the age of the population sample, as it is reported that although nonapatitic acid phosphate environments diminish with age, some fraction of acid phosphate ion is present at different levels of bone development[21]. The carbonate ion is present in bone mineral approximately 2–8% by weight[22] and its concentration is also age dependent[23]. The free ion possesses $D_{3h}$ molecular symmetry and therefore exhibits four normal vibration modes: $v_l$ (A'$_1$) (symmetric stretching), $v_2$ (A$_2$") (out of plane bending), and two doubly degenerate $v_3$, $v_4$ (E'), of which only the latter three are IR active. As is the case with the phosphate ion, the $v_1$ band is allowed when the symmetry lowers on the lattice carbonate location, but all the same, it is hidden beneath the $v_3$ phosphate band. The ion may occupy three different sites in bone mineral: in monovalent anionic sites substituting for the hydroxyl group (A-type), in trivalent anionic sites substituting for the phosphate group (B-type), or on the surface of bone apatite crystals at random locations. The detailed configuration of the carbonate ion (A or B) on either site in the lattice remains unknown, mainly due to the minute crystallite size of the apatite. On the other hand, A-type carbonate apatite features IR absorption near 879 $cm^{-1}$, while the B-type is at 871 $cm^{-1}$. A third band at 866 $cm^{-1}$ is assigned to labile (nonapatitic) carbonate, the concentration of which decreases as bone matures, although the overall carbonate content increases[24]. All these bands belong to the $CO_3^{2-}$ $v_2$ domain and they are observable by applying Fourier self-deconvolution to the spectra. The stronger IR band, $CO_3^{2-}$ $v_3$, is severely overlapped by intense absorption bands of organic material (Table 1), which makes this spectra area ineffective for further consideration. The $v_4$ fundamental shows a single very weak band at 669 $cm^{-1}$. As a result, only the $v_2$ mode is suitable for quantitative calculations of carbonates in the bone mineral. Absorption of the $HPO_4^{2-}$ group at the same region produces a broad band that can be neglected, since it has been shown that it does not interfere with the quantitative estimations involving the carbonate ion[25].

### IR Analysis

Overall mineral concentration can be estimated by the ratio of the integrated area of the $v_l$, $v_3$ phosphate absorbance bands to the integrated area of the protein Amide I absorbance band[26,27].

An alternative method to assess mineral aggregation is through the ratio of the $PO_4^{3-}$ $v_4$ area (500–650 $cm^{-1}$) to the integrated area (1600–1700 $cm^{-1}$) of the Amide I band[28]. Although analogous experimental results were obtained by either procedure, curve fitting analysis of the $v_4$ contour is more robust, since this can be fit to fewer components[29]. For this reason, content related the phosphate group was obtained from the $PO_4^{3-}$ $v_4$ spectral region.

The mineralization results are tabulated in the first column of Table 2, where there is a comparison between sex and bone sites. No significant disparities were observed between the two groups ($p > 0.05$), although standard deviation calculation showed that distribution of values for the male population is more scattered than the female population, resulting in a high coefficient of variation (10, 16, and 14% for tibia, femur, and forearm sections, respectively). To confirm the outcome of this method, simple area integrations of the $v_l$, $v_3$ curve was performed for all samples. No significant discrepancies were identified among the two groups or the three bone sites. By means of second derivative and curve fitting analysis of

**TABLE 2**
**Summary of Parameters Analyzed through Vibrational Spectroscopic Analysis**

| | Mineral | Labile $HPO_4^{2-}$ | Labile $PO_4^{3-}$ | Carbonate | Type A/Type B | Labile $CO_3^{2-}$/Type B | Collagen Maturity |
|---|---|---|---|---|---|---|---|
| Tibia | | | | | | | |
| M* | 2.75 ± 0.26 | 0.15 ± 0.02 | 0.09 ± 0.005 | 0.16 ± 0.02 | 0.16 ± 0.09 | 0.31 ± 0.03 | 2.79 ± 0.59 |
| F* | 2.66 ± 0.22 | 0.23 ± 0.02 | 0.08 ± 0.008 | 0.17 ± 0.02 | 0.15 ± 0.01 | 0.36 ± 0.05 | 2.37 ± 0.58 |
| Femur | | | | | | | |
| M | 2.88 ± 0.47 | 0.19 ± 0.03 | 0.08 ± 0.02 | 0.18 ± 0.03 | 0.15 ± 0.06 | 0.36 ± 0.06 | 2.81 ± 0.77 |
| F | 2.89 ± 0.12 | 0.28 ± 0.02 | 0.07 ± 0.002 | 0.16 ± 0.02 | 0.17 ± 0.02 | 0.39 ± 0.05 | 2.73 ± 0.90 |
| Forearm | | | | | | | |
| M | 2.93 ± 0.41 | 0.20 ± 0.08 | 0.08 ± 0.01 | 0.20 ± 0.05 | 0.14 ± 0.02 | 0.34 ± 0.04 | 2.30 ± 0.39 |
| F | 2.73 ± 0.12 | 0.23 ± 0.05 | 0.07 ± 0.002 | 0.17 ± 0.02 | 0.15 ± 0.01 | 0.38 ± 0.07 | 2.24 ± 0.37 |

* M, male; F, female.

the well-defined $PO_4^{3-}$ $v_4$ band, acid phosphate information was extracted from the sub-bands intensity ratio (Fig. 2). The wide contour was decomposed with the input of five Gaussian-shaped bands. In order to avoid inconsistencies between variable sets, we kept the baseline linear and the position of the band at 603 $cm^{-1}$ locked. All other parameters (peak center, width, and area) were left free. Two bands near 621 (±2) $cm^{-1}$ and a broad one at 532 (±8) $cm^{-1}$ are attributed to nonapatitic phosphate and acid phosphate, while the fundamental $PO_4^{3-}$ $v_4$ mode appears at 561 and 603 $cm^{-1}$, corresponding to crystalline apatitic environment along with the band at 580 $cm^{-1}$.

In accordance with the results of other groups[30], the band area at 621 $cm^{-1}$ remains practically constant, indicating 8–9% of labile $PO_4^{3-}$ in all cases (different population and/or bone site), showing that such reactive species might play an important role in the ion pool of the bone mineral throughout all ages[31]. The wide band centered between 524 and 540 $cm^{-1}$ is assigned to acid phosphate content where $HPO_4^{2-}$ ions are readily substitutable by carbonate groups. There is an increasing trend ($p = 0.08$) of the labile $HPO_4^{2-}$ from male to female population (Table 2). This finding is evaluated as particularly significant ($p < 0.01$) for tibia and femoral sites, and clearly demonstrates that female bone mineral features inherent intracrystalline disorder compared to that of males. This disorder in the female group may also be attributed to differences in the relative bone sizes of the two populations.

Carbonate concentration was measured by the $CO_3^{2-}$ $v_2$ (855–890 $cm^{-1}$) to Amide I ratio. Nonsignificant variations ($p > 0.05$) were observed involving either different populations or bone sites. The application of curve fitting of these two areas, respectively, provides valuable information about the substitution effect for the three types of carbonate and collagen cross-linking alterations related with the tensile strength and viscoelasticity of bone[32,33,34]. Using curve fitting procedures, we evaluated the relative site of carbonate substitution for all the homogenized bone samples. Second derivative analysis revealed three sub-bands for the $CO_3^{2-}$ $v_2$ contour located at 879, 871, and 866 $cm^{-1}$. The first two bands are assigned to apatitic locations of the carbonate ion in the two anionic sites of the structure normally occupied by phosphate and hydroxyl ions, respectively.

The results (Table 2) agree with those of other workers[35] that biological apatites are mainly B-type carbonate apatites with small fractions of A-type impurities (ca. 15%) and this ratio remains practically constant after the completion of bone mineralization. Thus, the prevailing substitution occurs for the phosphate ion and this is connected with an increased stability of the apatite itself[22]. Upon fitting, there is a slight shift of the labile $CO_3^{2-}$ sub-band to 862 $cm^{-1}$, indicating a sort of interaction and minor changes in the environment of the molecule. The ratio between labile and B-type carbonate was low and

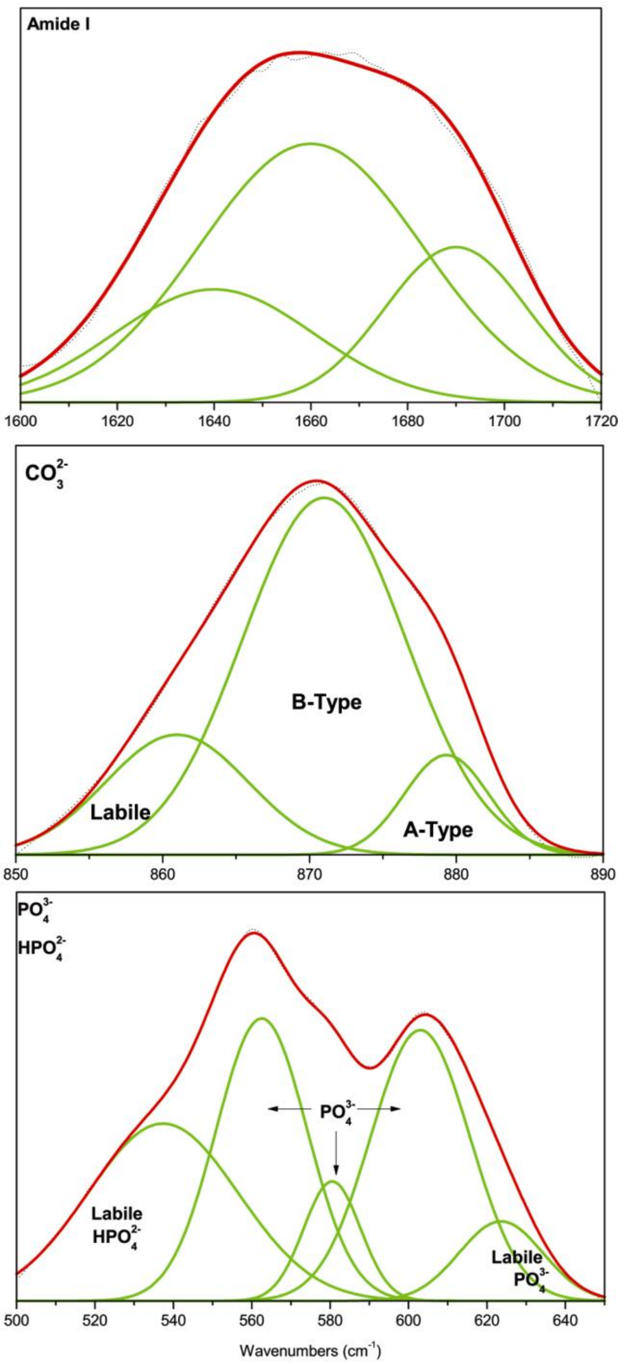

**FIGURE 2.** Typical curve fitting analysis of $PO_4^{3-}$ $v_4$, $CO_3^{2-}$ $v_2$, and Amide I IR bands.

analogous to mature bone mineral. There is an increasing tendency of this ratio between males and females that is statistically significant ($p < 0.05$). The means are slightly dissimilar among sites of the same sex, while naturally the peak area of the nonapatitic carbonate ion is generally decreasing upon bone maturation. We therefore conclude that in females, these carbonate species exist to an increased concentration, suggesting an inadequately structured area of the apatite crystal lattice.

There is a positive correlation ($p$ = 0.01) between the unstable carbonate location and the acid phosphate content found for all samples. Both ions exhibit a statistically significant increasing trend between populations and not among different bone sites. In Fig. 3, the linear regression plot of the two ions ($R$ = 0.83; slope = 0.59 ± 0.13; Y-int: 0.23 ± 0.03) is presented involving the medians for the two populations.

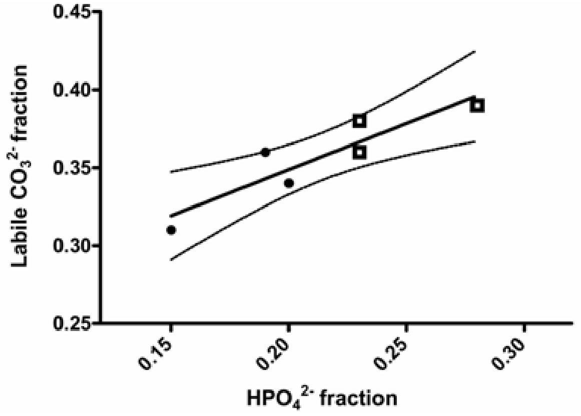

**FIGURE 3.** Linear relationship between labile carbonate and acid phosphate content of homogenized bone samples. The curves represent the 95% confidence band of the best fit line (● Male, □ Female).

The 1660/1690 $cm^{-1}$ ratio of the Amide I band has been related with the relative amount of nonreducible (mature) to reducible (immature) types of collagen cross-links[36]. This ratio has been shown to increase with collagen maturity and with the degree of mineralization. Fourier self-deconvolution as well as second derivative analysis was also utilized in this case to separate the two peaks. In accordance with our findings about mineral concentration, this ratio varies nonsignificantly ($p$ > 0.05) among specimens.

### EDX Measurements

Table 3 summarizes the results from the analysis of the bulk Ca/P atomic ratios of the cortical bone for different bone sites and sex.

**TABLE 3**
**Bulk Values of the Ca/P Ratio Measured by EDX Spectroscopy**

| *Bone Type* | *Mean Ca/P* | |
|---|---|---|
| | **Male** | **Female** |
| Tibia | 1.85 ± 0.10 | 1.84 ± 0.06 |
| Femur | 1.96 ± 0.11 | 1.93 ± 0.09 |
| Forearm | 1.95 ± 0.12 | 1.91 ± 0.13 |

If cortical bone contained only mineral (hydroxyapatite), a stable ratio Ca/P = 2.16 should exist. Nevertheless, cortical bone contains 30–40% organic matter, which affects the Ca/P ratio from that of pure hydroxyapatite, resulting in Ca-deficient apatite[37]. Medians involving all three bone sites do not vary significantly ($0.06 < p < 0.4$), while there is no statistical disparity between the two populations ($0.5 < p < 0.8$). Statistical differences comparing tibia and femoral sections are significant ($p = 0.04$) in accordance with previous findings[8,9] where synchrotron microCT was used. Other researchers have also noted differences in trabecular bone composition among skeletal sites[38]. Factors that may contribute to such variations are different bone cell populations, differential biomechanical tension, and local differences in the blood supply of bone. As trabecular is more metabolically active than cortical bone, it demonstrates higher divergence in composition between bone sites than in our case. The lower Ca/P ratio for the female group, albeit not particularly significant, is explained by the higher content of $HPO_4^{2-}$, as discussed in the previous section.

### CONCLUSIONS

It is very interesting to mention the discrimination observed by the different experimental procedures followed in this study. FTIR spectroscopy, which probes functional groups, classified the samples based on sex, while EDX spectroscopy, which probes elemental analysis, classified the samples based on bone sites. The discrepancies found among bone sites are probably due to variations of Ca concentration. Differences found between the two populations are likely due to instabilities of the carbonate and acid phosphate content originated from microscopic (molecular) or macroscopic (bone length) factors. In particular, the female population shows increased concentrations of the latter acidic group. The increase in monohydrogen phosphate and the replacement of some $PO_4^{3-}$ by $HPO_4^{2-}$ ions, even though it creates an anionic vacancy, are not primarily compensated by the removal of $Ca^{2+}$ from the lattice[39], as is evident from EDX measurements; according to IR spectra, this is most probably due to $CO_3^{2-}$ substitution following the increase of the unstable carbonate ions.

The deviation of the Ca/P ratio among bone sites follows the same trend with the variation of mineral content. The local nonapatitic environments of both carbonate and phosphate ions seem to play a crucial role in the ion pool of bone mineral. Upon maturation, these ions exist in noticeable concentration, enforcing the hypothesis of carrying out biological and mechanical functions by their enhanced reactivity[30]. The same argument stands for nonapatitic acid phosphate ions that, in general, decrease rapidly with carbonate accretion. Although many types of ionic substitutions in bone apatite lattice are critical to its crystallite size and dissolution rate, as far as the mature bone is concerned, the anionic substitution of B-type predominates. Maturity of collagen fibrils as extracted from FTIR data is in accordance with the age of the samples.

## References

1. Carrey, J.D. (2006) *Bones: Structure and Mechanics*. Princeton University Press.
2 Berillis, P., Emfietzoglou, D., and Tzaphlidou, M. (2006) Collagen fibril diameter in relation to bone site and to calcium/phosphorus ratio. *TheScientificWorldJOURNAL* **6**, 1109–1113.
3. Werner, C., Iversen, B.F., and Therkildsen, M.H. (1988) Contribution of the trabecular component to mechanical strength and bone mineral content of the femoral neck. An experimental study on cadaver bones. *Scand. J. Clin. Lab. Invest.* **48**, 457–460.
4. Lotz, J.C., Cheal, E.J., and Hayes, W.C. (1995) Stress distributions within the proximal femur during gait and falls: implications for osteoporotic fracture. *Osteoporos. Int.* **5**, 252–261.
5. Boskey, A.L., Goldberg, M., Kulkarni, A., and Gomez, S. (2006) Infrared imaging microscopy of bone: Illustrations from a mouse model of Fabry disease. *Biochim. Biophys. Acta* **1758**, 942–947.

6. Masuyama, R., Nakaya, Y., Katsumata, S., Kajita, Y., Uehara, M., Tanaka, S., Sakai, A., Kato, S., Nakamura, T., and Suzuki, K. (2003) Dietary calcium and phosphorus ratio regulates bone mineralization and turnover in vitamin D receptor knockout mice by affecting intestinal calcium and phosphorus absorption. *J. Bone Miner. Res.* **18**, 1217–1226.
7. Zaichick, V. and Tzaphlidou, M. (2002) Determination of calcium, phosphorus, and the calcium/phosphorus ratio in cortical bone from the human femoral neck by neutron activation analysis. *Appl. Radiat. Isot.* **56**, 781–786.
8. Speller, R., Pani, S., Tzaphlidou, M., and Horrocks, J. (2004) MicroCT analysis of calcium/phosphorus ratio maps at different bone sites. *Nucl. Instrum. Meth. A* **548**, 269–273.
9. Tzaphlidou, M., Speller, R., Royle, G., and Griffiths, J. (2006) Preliminary estimates of the calcium/phosphorus ratio at different cortical bone sites using synchrotron microCT. *Phys. Med. Biol.* **51**, 1849–1855.
10. Akesson, K., Grynpas, M.D., Hancock, R.G.V., Odselius, R., and Obrant, K.J. (1994) Energy-dispersive x-ray microanalysis of bone mineral content in human trabecular bone: a comparison with ICPES and neutron activation analysis. *Calcif. Tissue Int.* **55**, 236–239.
11. Bloebaum, R.D., Holmes, J.L., and Skedros, J.G. (2005) Mineral content changes in bone associated with damage induced by the electron beam. *Scanning* **27**, 240–248.
12. Morris, M.D. and Finney, W.F. (2004) Recent developments in Raman and infrared spectroscopy and imaging of bone tissue. *Spectroscopy* **18**, 155–159.
13. Movasaghi, Z., Rehman, S., and Rehman, I.U. (2008) Fourier transform infrared (FTIR) spectroscopy of biological tissues. *Appl. Spectrosc. Rev.* **43**, 134–179.
14. Petibois, C., Gouspillou, G., Wehbe, K., Delage, J.P., and Deleris, G. (2006) Analysis of type I and IV collagens by FT-IR spectroscopy and imaging for a molecular investigation of skeletal muscle connective tissue. *Anal. Bioanal. Chem.* **386**, 1961–1966.
15. Nakamoto, K. (2009) *Infrared Spectra of Inorganic and Coordination Compounds*. 6$^{th}$ ed. Wiley-Interscience.
16. Petra, M., Anastassopoulou, J., Theologis, T., and Theophanides, T. (2005) Synchrotron micro-FT-IR spectroscopic evaluation of normal paediatric human bone. *J. Mol. Struct.* **733**, 101–110.
17. Mkukuma, L.D., Skakle, J.M.S., Gibson, I.R., Imrie, C.T., Aspden, R.M., and Hukins, D.W.L. (2004) Effect of the proportion of organic material in bone on thermal decomposition of bone mineral: an investigation of a variety of bones from different species using thermogravimetric analysis coupled to mass spectrometry, high-temperature X-ray diffraction, and Fourier transform infrared spectroscopy. *Calcif. Tissue Int.* **75**, 321–328.
18. Taylor, M.G., Parker, S.F., Simkiss, K., and Mitchell, P.C.H. (2001) Bone mineral: evidence for hydroxy groups by inelastic neutron scattering. *Phys. Chem. Chem. Phys.* **3**, 1514–1517.
19. Wopenka, B. and Pasteris, J.D. (2005) A mineralogical perspective on the apatite in bone. *Mater. Sci. Eng. C* **25**, 131–143.
20. Posner, A.S. (1969) Crystal chemistry of bone mineral *Physiol. Rev.* **49**, 760–792.
21. Rey, C., Shimizu, M., Collins, B., and Glimcher, M.J. (1991) Resolution-enhanced Fourier transform infrared spectroscopy study of the environment of phosphate ion in the early deposits of a solid phase of calcium phosphate in bone and enamel and their evolution with age: 2. Investigations in the $v_3$ $PO_4$ domain. *Calcif. Tissue Int.* **49**, 383–388.
22. Bigi, A., Cojazzi, G., Panzavolta, S., Ripamonti, A., Roveri, N., Romanello, M., Suarez, K.N., and Moro, L. (1997) Chemical and structural characterization of the mineral phase from cortical and trabecular bone. *J. Inorg. Biochem.* **68**, 45–51.
23. Rey, C., Renugopalakrishnan, V., Collins, B., and Glimcher, M.J. (1991) Fourier transform infrared spectroscopic study of the carbonate ions in bone mineral during aging. *Calcif. Tissue Int.* **49**, 251–258.
24. Bohic, S., Rey, C., Legrand, A., Sfihi, H., Rohanizadeh, R., Martel, C., Barbier, A., and Daculsi, G. (2000) Characterization of the trabecular rat bone mineral: effect of ovariectomy and bisphosphonate treatment. *Bone* **26**, 341–348.
25. Rey, C., Collins, B., Goehl, T., Dickson, I.R., and Glimcher, M.J. (1989) The carbonate environment in bone mineral: a resolution enhanced Fourier transform infrared spectroscopy study. *Calcif. Tissue Int.* **45**, 157–164.
26. Paschalis, E.P., Betts, F., DiCarlo, E., Mendelsohn, R., and Boskey, A.L. (1997) FTIR microspectroscopic analysis of normal human cortical and trabecular bone. *Calcif. Tissue Int.* **61**, 480–486.
27. Faibish, D., Ott, S.M., and Boskey, A.L. (2006) Mineral changes in osteoporosis. A review. *Clin. Orthop. Relat. R*es. **443,** 28–38.
28. Huang, R.Y., Miller, L.M., Carlson, C.S., and Chance, M.R. (2003) In situ chemistry of osteoporosis revealed by synchrotron infrared microspectroscopy. *Bone* **33**, 514–521.
29. Rey, C., Shimizu, M., Collins, B., and Glimcher, M.J. (1990) Resolution enhanced Fourier transform infrared spectroscopy study of the environment of phosphate ions in the early deposits of a solid phase of calcium-phosphate in bone and enamel, and their evolution with age. I: Investigations in the $v_4$ $PO_4$ domain. *Calcif. Tissue Int.* **46**, 384–394.
30. Miller, L.M., Vairavamurthy, V., Chance, M.R., Mendelsohn, R., Paschalis, E.P., Betts, F., and Boskey, A.L. (2001) In situ analysis of mineral content and crystallinity in bone using infrared micro-spectroscopy of the $v_4$ $PO_4^{3-}$ vibration. *Biochim. Biophys. Acta* **1527**, 11–19.

31. Rey, C., Lian, J., Grynpas, M., Shapiro, F., Zylberberg, L., and Glimcher, M.J. (1989) Non-apatitic environments in bone mineral: FT-IR detection, biological properties and changes in several disease states. *Connect. Tissue Res.* **21**, 597–603.
32. Tzaphlidou, M. (2005) The role of collagen in bone structure: an image processing approach. *Micron* **36**, 593–601.
33. Oxlund, H., Barckman, M., Ortoft, G., and Andreassen, T.T. (1995) Reduced concentrations of collagen cross-links are associated with reduced strength of bone. *Bone* **17**, S365–S371.
34. Paschalis, E.P., Shane, E., Lyritis, G., Skarantavos, G., Mendelsohn, R., and Boskey, A.L. (2004) Bone fragility and collagen cross-links. *J. Bone Miner. Res.* **19**, 2000–2004.
35. Paschalis, E.P., DiCarlo, E., Betts, F., Sherman, P., Mendelsohn, R., and Boskey, A.L. (1996) FTIR microspectroscopic analysis of human osteonal bone. *Calcif. Tissue Int.* **59**, 480–487.
36. Paschalis, E.P., Verdelis, K., Doty, S.B., Boskey, A.L., Mendelsohn, R., and Yamauchi, M. (2001) Spectroscopic characterization of collagen cross-links in bone. *J. Bone Miner. Res.* **16**, 1821–1828.
37. Zaichick, V. and Tzaphlidou, M.(2003) Calcium and phosphorus concentrations and the calcium/phosphorus ratio in trabecular bone from the femoral neck of healthy humans as determined by neutron activation analysis. *Appl. Radiat. Isot.* **58**, 623–627.
38. Aerssens, J., Boonen, S., Joly, J., and Dequeker, J. (1997) Variations in trabecular bone composition with anatomical site and age: potential implications for bone quality assessment. *J. Endocrinol*. **155,** 411–421.
39. Legros, R., Balmain, N., and Bonel, G. (1987) Age-related changes in mineral of rat and bovine cortical bone. *Calcif. Tissue Int.* **41**, 137–144.
